# Report of the Salaries Committee of the College of Nursing

**Published:** 1919-06-14

**Authors:** 


					276 THE HOSPITAL June 14, 1919.
SALARIES AND CONDITIONS OF EMPLOYMENT
OF NURSES.
REPORT OF THE SALARIES COMMITTEE
OF THE COLLEGE OF NURSING.
A copy of the Report of the Committee appointed by the
Council of the College of Nursing has now been circulated
to institutions and to those who have supplied statistics to
the committee. The Council of the College has the Report
under consideration, and in due course will formulate sug-
gestions based upon it. Copies of the Report may be
obtained shortly from the publishers, Messrs. Eyre & Spottis-
woode, Ltd., East Harding Street, London, E.C., 4., or
from The Scientific Press, Limited, 28 and 29
Southampton Street, London, W.C.2, price 6d., or by
post 7d.
On page 253 et seq. of The Hospital for June 7 we
gave the substance of this Report which demonstrated to
the whole nursing profession how thoroughly, well the
College authorities had discharged their responsible duty
in appointing and selecting so able a Committee fully
to accomplish the work entrusted to them. In con-
tinuing to give further portions of the report and tables
of the Salaries and Employment Committee of the Col-
lege of Nursing, we have now the duty to set out the
tables showing (I.) the scale of minimum salaries recom-
mended; (II.) the actual salaries in institutions; (III.)
the average salaries paid in hospitals with more than
500 beds in daily occupation; (IV.) the average salaries
paid in hospitals with 300 to 500 such beds; (V.) the
average salaries paid in hospitals with 200 to 300 such
beds; (VI.) the average salaries paid in hospitals with
100 to 200 such beds; (VII.) the average salaries paid in
hospitals with 50 to 100 such beds; (VIII.) the average
saJaries paid in hospitals with 25 to 50 beds; and,
finally, table (IX.), the average salaries paid in hospitals
with less than 25 daily occupied beds.
We propose to give the conclusion of the Report in our
next issue. To-day we venture to give a word of en-
couragement to every nurse and all interested in this
important Report, and the subjects with which it deals,
by. pointing out that the tables and figures they contain,
though they may look formidable when the eye first takes
them in, -will, with the aid of quiet pemsal and the
determination to understand and master this portion of
thei Report, prove invaluable in many ways. The facts
they contain will give every nurse important information
calculated to equip her happily to take part in the battle
of life which lies before each in the future. We are not
all masters of figures, it is true, but everyone of us should
make a point of becoming accustomed to their use and
meaning, for then many, worries wise people avoid will
cease to trouble them. So equipped, every nurse may
pursue life's journey, pale, calm, unmoved, amid the
storms of life, and one day wear
" Patience?
Of whose soft grace, I have her sovereign aid,
And rest myself content."?Shakespeare.
May every reader who studies the tables go on and
prosper, having a heart full of gratitude for the able
Committee to whom each and all are indebted for the
information in this Report, and abiding faith in the
splendid future of the College under the direction of its
democratic Council, elected with judgment and confidence
by the overwhelming majority of fully-trained British
Scale of Salaries.
The rates of salaries recommended by the Committee as
shown in the accompanying scale apply to: (1) General hos-
pitals; (2) Poor-Law institutions; (3) hospitals for infec-
tious diseases; (4) hospitals for special diseases; (5) con-
sumptive sanatoria; (6) private nursing homes.
Hospitals for Incurables.?One grade lower in the scale of
salaries than is shown by the average number of beds occu-
pied daily.
Convalescent Homes, Epileptic Colonies, and Homes for
Defectives??Two grades lower in the scale than is shown by
the average number of beds occupied daily.
In the case of convalescent homes, homes for defectives,
epileptic colonies, and hospitals for incurables no proba-
tioners should be taken, and the minimum staff of sisters
and nurses laid down as being necessary in nursing insti-
tutions does not apply. The salaries also are suggested as
being applicable only to hospital-trained nurses of a recog-
nised training school.
The salaries shown on the scale are the minimum salaries
recommended in normal times, as allowing a nurse a suffi-
cient income to live upon, even if she does not rise to the-
better positions offered in the profession. At the same time
the hospitals will offer better salaries to the more highly
qualified nurse. In times of high prices the salaries should
be augmented temporarily.
It is not thought desirable to fix maximum salaries, but
indication has been made of the annual increments applicable
to each class.
If nurses live out from the hospitals and no nursing home
is provided, then a sum of not less than ?45 a year should
be allowed for rooms, and not less than ?35 a year for meals
taken outside.
Where district nurses and others are paid a resident
salary, allowances being made for board and lodgings, a
.house or rooms should, not be provided unless attendance
is provided as well.
Table I.
Scale of Minimum Salaries Recommended.
Average
Number of
Beds Occu-
pied*
Above 500
300-500
200-300
100-200
50-100
25- 50
Under 25
Matron.
?
350
300
250
200
150
120
100
Assist.
Matron
?
130
120
100
? or
Home
Sister.
?
100
90
80
80
75
60
60
Night
Supt.
?
100
90
80
80
75
"Ward Sister.
30 Beds!
?
75
75
75
75
75
20 Beds|
?
60
60
60
Staff
Nurse.
?
60
60
60
60
?1
50
50
50
50
50 ?
50
50
* To be taken over a period of not less than three years
pre-war.
PROBATIONERS.
Children's Hospitals and Hospitals for Infectious Diseases.
At the age of 18  ?12
19   ?12
20 ...  . ?15
21  ?18
General Hospitals.
1st year    ... ?18
2nd year      ?22
3rd year  ?30
For each special certificate desired by the hospitals other
than the three years' general training certificate ?10 a year
at least extra.
Matrons to rise by ?10 a year to the maximum salary.
Assistant matrons to rise by ?10 a year to the maximum
salary.
Home sisters, night superintendents, ward sisters, staff
nurses, charge nurses, all rising by ?5 a year to the
maximum salary.
Statistics.?The averages shown in the accompanying lists
do not include war bonuses, which are still being _ pasd in
some hospitals, and in nearly all Poor-Law institutions;
neither do they include pension premiums paid by certain
hospitals.
June 14, 1919. THE HOSPITAL 277
Salaries and Conditions of Employment? {cont.).
Table II.
Salaries in Institutions.
The salaries range from?
Institutions.
Matron .
Assistant Matron
Home Sister .
JTight'Superintendent
"Ward Sister .
Other Sisters .
StafE Nurses .
Probationers?
4
3
2
1
Above
*500. I
?
450-80,
100-50
100-40
100-36
55-32
200-35
45-26
32-18
40-10
26- 6
22- 5-
300 to
500.
?
250-100
100- 50
70- 40
75- 45
60- 35
100- 35
50- 30
42?- 22
30- 17
26- 12
22- 10
200 to I
300.
?
250-45
120-55
80-40
80-35
55-35
100-45
55-28
40-25
30-18
25-14
22-10
100 to
200.
200-60
100-45
75-40
65-40
60J-35
100-40
45-24
40-18
30-16
26-14
24-10
50 to ,
100.
?
200-55
75-40
65-32
65-32
65-25
84-40
45-20
36-15
35-10
30- 8
26-
25 to |
50.
?
200-45
72-40
90-50
60-40
60-30
55-45
50-21
40-20
30-12
26-10
22- 5
Less
than25
?
150-35
75-40
70-40
72-28
50-45
55-20
30-18
32-14
24-12
27- 5
* These figures show the average number of beds in daily
occupation
Table III.
Average Salaries Paid in Hospitals of more than
500 Beds in Average Daily Occupation.
Institutions
London General
ProvincialGeneral
Scottish General
London Poor Law |
Infirmaries
Prov. Poor Law
Infirmaries
Scottish Fever
Hospitals
Irish Poor Law
Infirmaries
Suggested
No.
considered
Matron
?
310
260
250
126
156
183
120
Assistant
Matron
?
78
100!
87
GO
70*|
75
65
130'
I Home
| Sister
?
65
90
65
47
57*|
57
61
Night
Supt.
?
621|
65
70
44
53
75
100
"Ward
Sister
73 I
? I
Other
Sisters
?
52-108|
45-80
GO
59-60
51-75
50-55
Staff
Nurses
?
37
374!
40
30
354]
34
40
Probationers
18
50
3
?
25
24
26
224
24
30
?
19
19|l
21
19
18*1
18
Table IV.
Average Salaries Paid in Hospitals with 300 to
500 Beds in Average Daily Occupation.
Institutions
London General
Provincial General
London Poor Law,
Infirmaries.
Provincial Poor
Law Infirmaries
Scottish General
and Poor Law
Infirmaries
Suggested
No.
considered
Matron
?
(152
175
118
125
118
300,
Assistant
Matron
?
62J
78
60}
69
68
120
Home
Sister
90
Night
Supt.
90
Ward Sister
?
42
49
42J
46
In
charge I
30
beds?|
?75,
20
beds?
?60
Other
Sisters
?
35-55
55J-72
47
50-55
46-601
Staff nurses
Probationers
30
22]
1.
?
15}
14
14
14
13
18
Table V.
Average Salaries Paid in Hospitals with 200 to
300 Beds in Average Daily Occupation.
Institutions.
Provincial General]
Provincial Poor Law|
Infirmaries.
Scottish Fever and
General.
London Special _ .
ProvincialInfectious|
Suggested .
No.
considered!
Matron.
?
149
115
200
124
250
Assistant
Matron.
100
Home
Sister.
?
57
48i[
60
57i|
59
Night
Suet.
?
53
46
Ward
Sister
?
46
43
47*
47i
43
Other
Sisters
?
52-72
42i
52i
Stall
Nurses.
?
374|
37
40
60
Probationers ]
50
?j a>
V*
3 g
ofc 1
24 J
30
30
2 ,
? i
im
16
25J
20;
22 J |
22
, 1
\ ?
12
.n
20J
i1?>
'19
13
Table VI.
Average Salaries Paid in Hospitals of 100 to
200 Beds in Average Daily Occupation.
Institutions.
London General
Provincial General
Scottish Fever and
General.
Irish General
Provincial Poor
Law ,
Provincial, In-
fectious
London, Special
London, Incurables
Provincial, Special.
Suggested .
No.
considered
?
132
133
141
140
75||
130
i47i:!
115
98
200
Assistant
Matron.
80o
Home
Sister.
?
524!
52
40
55J
67*
58
48
40
r80
Night
Supt.
?
43
!??
40
51
48
80
Ward
Sister.
?
39
47
50
47
42i|
48
45
50
43
75
Other
j Sisters.
?
50-60
57-72
55
47-55
62-100|
50
I Staff
| Nurses.
&
Ml
35"
35
45
34
35
30
30
24
Probationers.
4
?
28 :
31
31?
18 '
=3 SS
4
3.
?
21
22
26
154|
21
27ij
23
25
19
30
2
?
18
18
22
12
16i|
24
19J|
22
14i|
22
I
1
?
11
13
18
n
13
21
17
20
10J
Is]
Table YII.
Average Salaries Paid in Hospitals with average 50 to
100 Beds Occupied Daily.
Institutions
London Generalj
Scottish General
Provincial,
General
London, Special
Provincial, Fever
Provincial, Special
Provincial, Poor
Law
Irish Hospitals
Irish, Special
Hospitals
Epileptic Colonies
Provincial,
Incurabks
Suggested ?
No. con-
sidered
Matron
?
127
96
100
109
96
96}
80
110
80
80
92}
150
Assistant!
Matron
75 o i
Home
Sister
45
Night
Supt.
?
42J|
52
44
45J|
49
47i|
50
4011
40
35
"Ward
Sister
?
43
47
43
42
44
45
45
39
37J
Other
Sisters
57*
56-64
50
49
50-55,
44*
50
Staff
Nurses
?
37
39
33
|
37 !
32 I
294;
31i
20
Probationers
4
?
28
25
28
27
284|
30
16*
26 |
?
23
20
20
24
26
23FI
274|
11
20
22
30
2
f ? !
[19 ;
15|!
16 1
19
22JI
19
23
8i|
13
24
19
22
1
?
a
12
16
20
14
18J
8
' 9
20
15
18
(Continued on page 278.)
' \
278  THE HOSPITAL June 14, 191$.
SALARIES AND CONDITIONS OF EMPLOYMENT?(concluded from p. 277).
Table VIII.
Average Salaries Paid in Hospitals of 25 to
50 Beds in Average Daily Occupation.
Institutions
Provincial, General
Scottish, General .
Irish, General
London, Special .
Provincial, Special
Provincial, Fever .
Suggested.
No.
considered
Matron
100
69
111
84
120
Assistant
Matron
?
52i
60
60 o
Home Sister
?
564
60
70
54
50
55
r60
Night
Supt.
?
47
40
40
48
52i
Ward
Sister
?
45J
50 ?
44
48
44J
n
Other Sisters
Stafi
Nurses
?
3311
421
351
30
32i|
32
Probationers
4
?
35
33i
18
20
30
25
a 2
.2 v*
g ho
J-* \?
Eh?5?
Table IX.
Average Salaries Paid in Hospitals with Average
of less than 25 Beds Occupied Daily
Institutions
Provincial, Fever
Irish Hospitals
London, Special
London, General
Scottish, General
Provincial, General
Provincial, Special
Suggested; . .
No.
considered.
Matron.
?
81
60
69
99
62?
100
Assistant
Matron.
75
Home
Sister.
Night
Supt.
Ward
Sister.
?
42
36
44
?*|
46
47
Other
Sisters.
47
Staff
Nurses.
?
30
37
31
35
44i
36J
27
60
Probationers
18:
28!:
27
16
26
21
19
214
20
2
?
22
15
19
17
18
I!11
1
?
19
11
15
14
141
13
14
Trained Nurses
?50

				

